# Trench Nephritis: Its Later Stages and Treatment

**Published:** 1917-11

**Authors:** 


**Trench Nephritis : Its Later Stages and Treatment.** By J. Mitchell CLARKE, Lt. C. R. A. M. C. (T.). Abstracted from the *British Medical Journal*, Aug. 25, 1917.

C. considers 74 cases observed in a base hospital service. Most of these had a previous history of acute infectious diseases. In some, " trench " nephritis began immediately after such diseases.

The affection nearly always began with a sudden attack of pain in the back accompanied by dyspnea and edema of the face and limbs. Slight hematuria was frequent. Uremic fits occurred in only one of the 74 cases. The systolic and diastolic blood pressures were generally increased in proportion to the severity of the disease. Retinal changes were not common. When present, they were usually associated with the severe form of the disease. Urine generally showed a large quantity of albumin early in the disease followed by a rapid decline in amount. Casts were usually present.

Most cases did well : 3 out of 68 died. 8 were discharged slightly improved but unfit for service. 11 still showed evidence of kidney disease. 6 were discharged well enough to do light work. 32 were completely restored. C. believes that patients leaving the hospital without abnormal urinary signs, and with blood pressure normal, are permanently cured.

Treatment : A strict rest in bed, and diet beginning with milk followed by small amounts of bread, rice, butter, potatoes, greens, jam, and occasionally fruit.

C. believes the pathology of the condition is that of a diffuse nephritis affecting chiefly the convoluted tubules.

